# One-Year Outcome of an Ongoing Pre-Clinical Growing Animal Model for a Tissue-Engineered Valved Pulmonary Conduit

**DOI:** 10.3390/jcdd11060179

**Published:** 2024-06-12

**Authors:** Martin Schweiger, Bernard Krüger, Alexandra Malbon, Thea Fleischmann, Miriam Weisskopf, Thomas Frauenfelder, Frithjof Lemme, Nikola Cesarovic, Walter Knirsch, Michael Hübler

**Affiliations:** 1 Department of Congenital Cardiovascular Surgery, Pediatric Heart Center, University Children’s Hospital Zurich, 8032 Zurich, Switzerland; 2 Children’s Research Center, University Children’s Hospital Zurich, 8032 Zurich, Switzerland; walter.knirsch@kispi.uzh.ch; 3 Division of Cardiac Anesthesia, Institute of Anesthesiology, University Hospital Zurich, 8091 Zurich, Switzerland; bernard.krueger@usz.ch; 4 Department of Anesthesia, University Children’s Hospital, 8032 Zurich, Switzerland; 5 The Royal (Dick) School of Veterinary Studies and the Roslin Institute, University of Edinburgh, Easter Bush Campus, Midlothian EH8 9YL, UK; alexandra.malbon@ed.ac.uk; 6 Division of Surgical Research, University Hospital Zurich, 8091 Zurich, Switzerlandmiriam.weisskopf@usz.ch (M.W.); 7 Institute of Diagnostic and Interventional Radiology, University Hospital Zurich, University Zurich, 8091 Zurich, Switzerland; 8 Congenital and Pediatric Heart Surgery, Children’s Heart Clinic, University Heart Center, University Medical Center Hamburg-Eppendorf, 20251 Hamburg, Germanym.huebler@uke.de (M.H.); 9 German Heart Center, Charitee, 13353 Berlin, Germany; 10 Division of Pediatric Cardiology, Pediatric Heart Center, University Children’s Hospital Zurich, 8032 Zurich, Switzerland

**Keywords:** tissue engineering, pulmonary valve, pulmonary valved conduit, remodeling

## Abstract

*Objectives*: A self-constructed valved pulmonary conduit made out of a de-cellularized porcine small intestinal submucosal extracellular matrix biological scaffold was tested in a chronic growing lamb model. *Methods*: The conduit was implanted in pulmonary valve position in 19 lambs. We monitored clinical, laboratory, and echocardiographic findings until 12 months after surgery. In two animals, euthanasia was planned at nine and twelve months. Pre-mortem chest computed tomography and post-mortem pathologic work up were performed. Data are presented as frequency and percentage, median and range, or mean and standard deviation. *Results*: Twelve (63.2%) animals survived the perioperative period. Three unexpected deaths occurred during the follow-up period: one due to aspiration pneumonia at 23 days after surgery, and two due to early and late infective endocarditis of the conduit at 18 and 256 days. In the two animals with planned scarification, the pre-mortem CT scan revealed mild or no calcification within the conduit or valve leaflets. In the echocardiographic examination at 12 months, peak and mean systolic pressure gradients across the conduit valve were 6.5 (3–21) mmHg and 3 (2–12) mmHg, while valve regurgitation was none (n = 2), trivial (n = 5), moderate (n = 1), or severe (n = 1). No clinical or laboratory signs of hemolysis were seen. After 12 months of follow-up, the animals’ body weights had increased from 33 (27–38) kg to 53 (38–66) kg (*p* = 0.010). *Conclusions*: Implantation of a valved pulmonary conduit in our growing lamb model was feasible. Infective endocarditis of the implanted valved conduit remained a significant complication.

## 1. Background

In children suffering from congenital heart defects such as common arterial trunk, pulmonary atresia with ventricular septal defect, or in children undergoing a Ross procedure, a long-lasting functionality of a connection with an integrated valve implanted between the right ventricle (RV) and the pulmonary artery (PA) is of utmost importance. Limitations of currently used valved conduits for this purpose, namely Contegra^®^ or cryopreserved homografts [1,2,3,4,5], have led to an ongoing search for alternatives. Recently, the development of tissue-engineered (TE) heart valves may open up new aspects because of their promising ability to (1) provide an appropriate and sustaining hemodynamic profile, (2) exhibit low thrombogenic properties, and (3) include the potential to grow [6].

We evaluated a self-constructed TE valved conduit (CorMatrix^®^ Cardiovascular, Inc., Atlanta, GA, USA) in orthotopic RV to PA position in a growing large animal model. We aimed to analyze whether the valved conduit remains stable in function, grows during the follow-up period, and finally whether TE patch material would be replaced and colonized by host tissue on histological examination after necropsy. The study protocol was published previously [7]. Here, we report on mid-term results after 12-month follow-up focusing on animal growth, conduit valve function determined by transesophageal echocardiography, course of laboratory markers, and results of CT and pathological examination at time of necropsy in planned or unplanned death of animals. The hypothesis was to find a functional valved conduit with a histologically proven endothelialization and no laboratory signs of hemolysis.

## 2. Methods

Animal housing and experimental protocols were approved by the Cantonal Veterinary Office, Zurich, Switzerland (License No. ZH 284/14), according to the Swiss Animal Protection Law. Housing and experimental procedures also conformed to European Directive 2010/63/EU of the European Parliament, of the Council of 22 September 2010 on the Protection of Animals used for Scientific Purposes, and to the Guide for the Care and Use of Laboratory Animals (2010/63/EU, 2010; Balinger et al., 2011).

### 2.1. Valved Conduit (Self-Created)

The creation of the valved conduit out of a commercially available de-cellularized porcine small intestinal submucosal extracellular matrix (ECM) biologic scaffold (Cormatrix^®^ Patch; CorMatrix^®^ Cardiovascular, Inc., USA), operative technique, and post-operative follow-up was described by our study group previously [7].

Briefly, the longitudinal side was divided into three equal parts mimicking the later created commissures using a 1:1.2 ratio between the conduit diameter and the leaflet height. Then, the leaflet part was folded, and the attachments of the commissures were reinforced with a pledged suture. Finally, the conduit was closed with a double running suture (Supplementary Data; Figure S1). The diameter of the conduit was tailored according to the diameter of the native pulmonary annulus of the lambs as pre-operatively assessed by transesophageal echocardiography.

### 2.2. Surgical Procedure and Post-Operative Care

Implantation of the valved conduit was performed in a beating heart supported by cardiopulmonary bypass (CPB). The native pulmonary valve trunk of the lamb with all pulmonary valve leaflets was resected in toto. The length of the conduit was tailored according to the length of the resected pulmonary trunk. Before closure, a chest tube was inserted and removed before extubation. After surgery, the animals were extubated in the operating room before transfer to the stable. For five days, all animals received acetylsalicylic acid (100 mg po, qd) and antibiotics (penicillin 35,000 IU per kg iv, bid, and gentamycin 4 mg per kg iv, qd). Further, all animals received external heat support, warmed fluids, and electrolyte supplementation. Each sheep was kept under close surveillance until respiratory rate, SpO_2_, and core body temperature were within the normal range.

### 2.3. Follow-Up Protocol

After one month, the valved conduit function was evaluated by transesophageal echocardiography. The lambs were then transported back to the farm for long-term husbandry and surveillance. The detailed echo protocol and the follow-up examinations were reported previously by this study group [7]. Follow-up controls included a clinical, laboratory, and echocardiographic examination and were scheduled at regular time intervals: once within the first post-operative month then at 3, 6, 9, and 12 months. Two experienced specialists performed and analyzed the 2D and 3D transthoracic and transesophageal echocardiography. Pulmonary conduit function was evaluated using Color Doppler and pulse wave/continuous wave (CW) Doppler in the area of the valve, the suture lines, the main pulmonary artery, and main side branches. Based on recommendations applied in humans [8,9], conduit valve regurgitation was labeled grade 0 for absent, grade 1 for trivial, grade 2 for mild, grade 3 for moderate, and grade 4 for severe according to valve morphology, regurgitation jet width, CW Doppler density, and RV size. Conduit valve stenosis was graded none, mild, moderate, or severe according to valve morphology and mean and systolic pressure gradients derived from valvular blood flow velocity.

### 2.4. Necropsy

When the planned endpoint was reached, the animals were scheduled for a CT scan before euthanasia and full post-mortem examination. The complete heart together with the great vessels were explanted. The heart chambers and outflow tracts were opened and visually inspected whilst preserving the integrity of the valved conduit leaflets. All explanted material was fixed in neutrally buffered formalin 10% to allow further processing for histologic examination. Tissues were then trimmed and routinely processed through to paraffin wax. Sections of 2 µm were cut onto glass slides for routine hematoxylin and eosin staining. Special staining and immunohistology (IH) were then performed on representative sections of valve and conduit. Stains included HE, whilst IH staining was performed for CD31 (endothelia), Iba-1 (macrophages), and vimentin (mesenchymal cells).

### 2.5. Study Population

For the chronic trial, a total of nineteen Swiss White Mountain ewe lambs with a body weight of 30 (24–38) kg were operated on. Of these, seven (36.8%) lambs died during the perioperative period. Two animals died in the OR and five within 48 h of surgery (Table 1). This report focuses on the surviving twelve animals.

### 2.6. Statistical Analysis

Data are presented as mean ± standard deviation (SD), median and range, or frequency and percentage. A repeated measures analysis of variance (ANOVA) was utilized to assess the impact of time after surgery on weight, pulmonary annulus, mean pulmonary gradient, and pulmonary regurgitation. Eta-squared (η^2^) was calculated as a metric to assess the magnitude of the effect (effect size) of time on the dependent variable. Prior to ANOVA, the assumption of sphericity was evaluated using Mauchly’s Test, which examines whether the variances in the differences between all combinations of related groups are equal. Overall significance level was set to 5%.

Figures were constructed using Excel (Version 2021, Microsoft Corp., Redmond, WA, USA).

## 3. Results

Out of nineteen lambs, twelve (63.2%) recovered from the operation and formed the study population. Up to 12 months follow-up, two lambs underwent planned sacrifice at 9 and 12 months per study protocol and three lambs died unexpectedly. Seven sheep remain for further observation.

Demographic data, surgical details, and outcome of the study cohort are displayed in Table 2. Compared to surgery non-survivors, OR time and CPB time showed a trend toward lower values in animals surviving surgery, but this did not reach statistical significance (*p* = 0.6 and *p* = 0.1, respectively).

### 3.1. Growth and Functionality of the Valved Conduit

**Figure 1:**
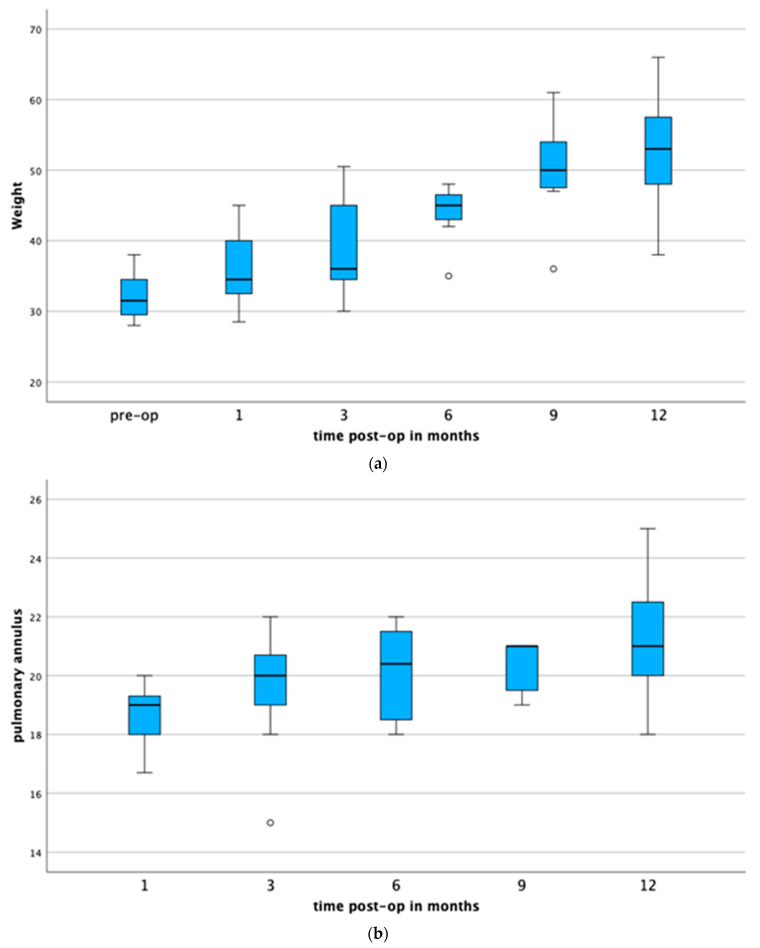
(**a**) Animal growth curve. There was a significant difference between pre-op, 6 months (*p* = 0.002), 9 months (*p* = 0.008), and 12 months (*p* = 0.010). Only animals were included that survived first 30 post-operative days. (**b**) Size of the valved pulmonary conduit measured at the level of the leaflet insertion. A repeated-measures ANOVA showed a significant difference between 1 and 9 months after surgery (*p* = 0.011). Only animals were included that survived the first 30 post-operative days.

The animals’ body weights increased after twelve months from 33 (27–38) kg to 53 (38–66) (Figure 1a). The outcome of time on weight was significant at the 0.05 level, F(1.57, 10.96) = 19.53, *p* < 0.001, partial η^2^ = 0.736. Pairwise post hoc tests using Bonferroni correction for multiple testing show a significant difference between pre-op and 6 months (*p* = 0.002), pre-op and 9 months (*p* = 0.008), pre-op and 12 months (*p* = 0.010), and 1 month and 12 months (*p* = 0.045).

The diameter of the valved conduit increased after twelve months by 11% (18 ± 1.2 mm to 20 ± 2.2 mm) (Figure 1b). The outcome of time on pulmonary annulus was significant at the 0.05 level, F(1, 7) = 10.12, *p* = 0.015, partial η^2^ = 0.591. Pairwise post hoc tests using Bonferroni correction for multiple testing show a significant difference between 1 month post-op and 9 months (*p* = 0.011).

**Figure 2:**
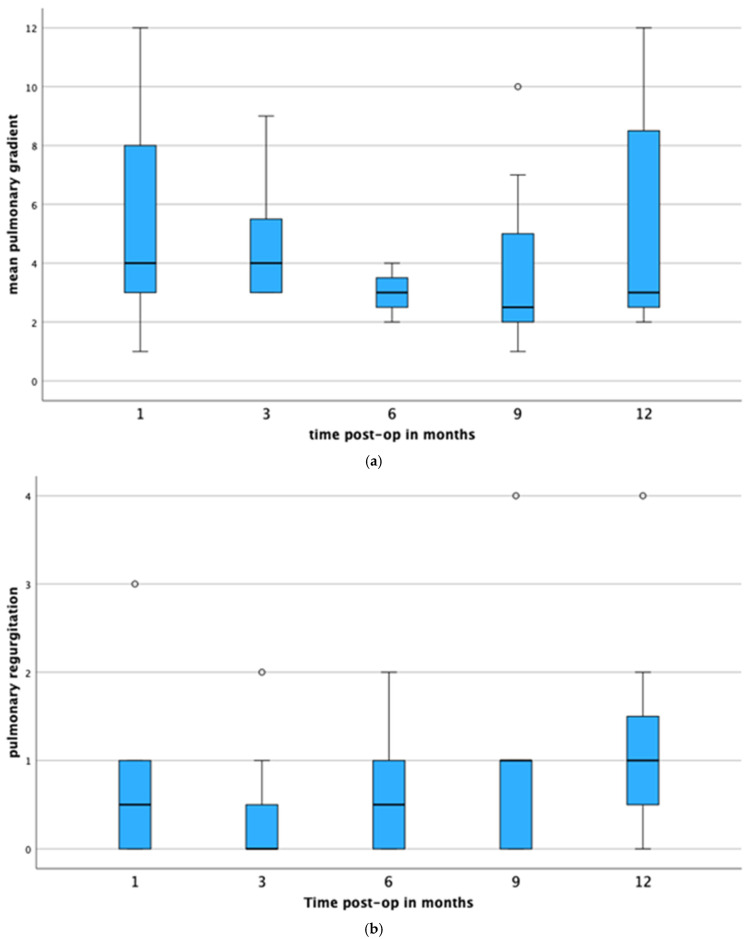
(**a**) Mean gradients across the valved pulmonary conduit during the 12-month follow-up period. Boxplots show minimum, first quartile, median, third quartile, and maximum values. (**b**) Valved pulmonary conduit regurgitation throughout the study period. Black line shows median, and gray bars show minimum and maximum pulmonary regurgitation grades. Grade 0: none, grade 1: trivial, grade 2: mild, grade 3 moderate, and grade 4: severe.

Functional evaluation of the valved pulmonary conduit by transthoracic and transesophageal echocardiography revealed no more than a mild valvular stenosis in surviving animals after 12 months. Mean and peak systolic Doppler gradients were 3 (2–15) mmHg and 6.5 (3–21) mmHg, respectively. The highest peak systolic gradient measured throughout the follow-up period was 21 mmHg (animal #16) at nine months. Mean gradients across the valved pulmonary conduit during the follow-up period are shown in Table 3. There was no statistic significant difference (*p* = 0.234), and the outcome of time on systolic Doppler gradients was not significant at the 0.05 level, F(4, 28) = 0.614, *p* = 0.656, partial η^2^ = 0.081 (Figure 2a). In five animals, echocardiography revealed signs of mild valve calcification over time: three months after surgery in one animal (#16) and 12 months after surgery in four animals (#5, #15, #22, #23). Out of the eight surviving animals at 12-month follow-up, mild valved pulmonary conduit calcification was present in four (50%) animals (Table 2).

No more than mild-to-moderate conduit valve regurgitation was observed up to 12 months follow-up except in one case (animal #14). The course of conduit valve regurgitation over time is depicted in Table 3. The outcome of time on valved conduit regurgitation was not significant at the 0.05 level, F(4, 28) = 1.89, *p* = 0.141, partial η^2^ = 0.212 (Figure 2b). In the animal with later calcification, the prolapse of one leaflet resulted in a severe conduit valve regurgitation nine months after surgery.

### 3.2. Laboratory Parameters

In the literature, mean (5–95% range) reference values of lactate dehydrogenase (LDH), hemoglobin, and white blood cell count in sheep are described as 995 (743–1325) U/L, 115 (9.8–13.2) g/L, and 7.3 (4.5–11.4) (103/μL), respectively [10,11]. The course of laboratory markers in this study is demonstrated in Table 4. The values of LDH were elevated immediately after surgery with 1215 U/L (915–1861) and remained well above 1000 U/L throughout the follow-up period with a value of 1121 U/L (951–1514) at 12 months. Hemoglobin values were stable throughout the observation period with 110 (88–115) g/L at 1 month after surgery and 108 (93–113) g/L at 12 months. Clinically, no signs of hemolysis were observed in the animals, and no blood transfusions were given at any time during the trial. The white blood cell count was not elevated throughout the study.

### 3.3. Necropsy and Pre-Mortem CT Scans

Two sheep were sacrificed according to the study protocol at 9 months (#16) and 12 months (#14) after surgery.

**Figure 3:**
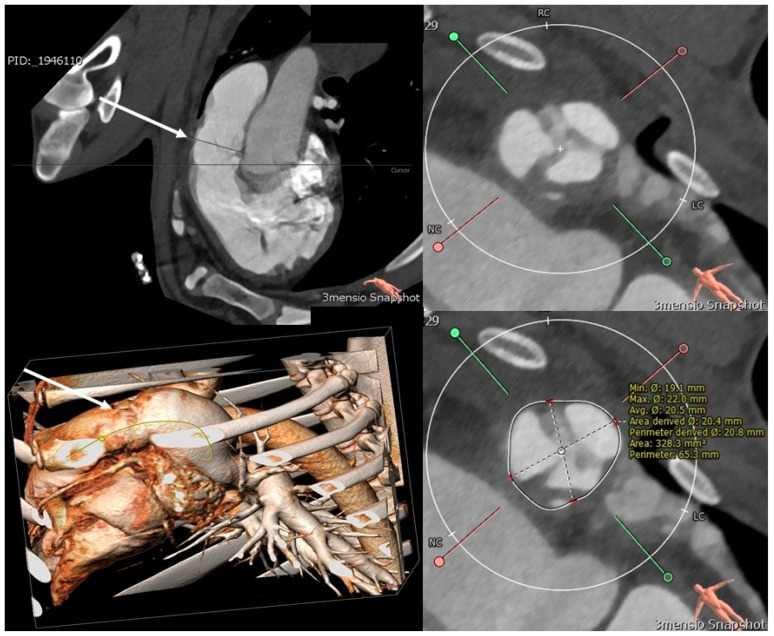
CT scan at time of euthanasia: Upper left image shows the valved conduit in long axis, and lower left image shows a 3D reconstruction of the valved conduit in situ (white arrows). Images on the right side show the conduit valve in short axis with three leaflets (**upper image**) and measurements (**lower image**).

In animal #16, the CT scan prior to euthanasia revealed mild signs of calcification close to the insertion of the valve cusp leaflets. The conduit measured 30 mm in length. The leaflets were slightly thickened (Figure 3). The dimensions of the conduit were 19.5 × 21.4 mm (area: 315 mm^2^, circumference: 63.9 mm) at the level of the native pulmonary annulus (proximal anastomosis) and 18.2 × 20.9 mm (area: 343 mm^2^, circumference: 68 mm) at the level of the conduit leaflets. There was a thickening of the right ventricular myocardium (6 mm) and a dilatation of the native pulmonary trunk (maximal diameter: 28 × 37 mm). The lung showed slight subpleural changes in the left posterior lobe.

In animal #14, there were no signs of calcification of the conduit and the valve leaflets appeared normal. The length of the conduit measured 28 mm. The dimensions of the conduit were 22.7 × 26.9 mm (area: 458 mm^2^, circumference: 77.5 mm) at the level of the native pulmonary annulus (proximal anastomosis) and 22 × 29 mm (area: 497 mm^2^, circumference: 80.5 mm) at the level of the leaflets. The right ventricular myocardium measured 4 mm, and the native pulmonary trunk measured 23 × 37 mm. The lung parenchyma appeared normal.

### 3.4. Unexpected Deaths

**Figure 4:**
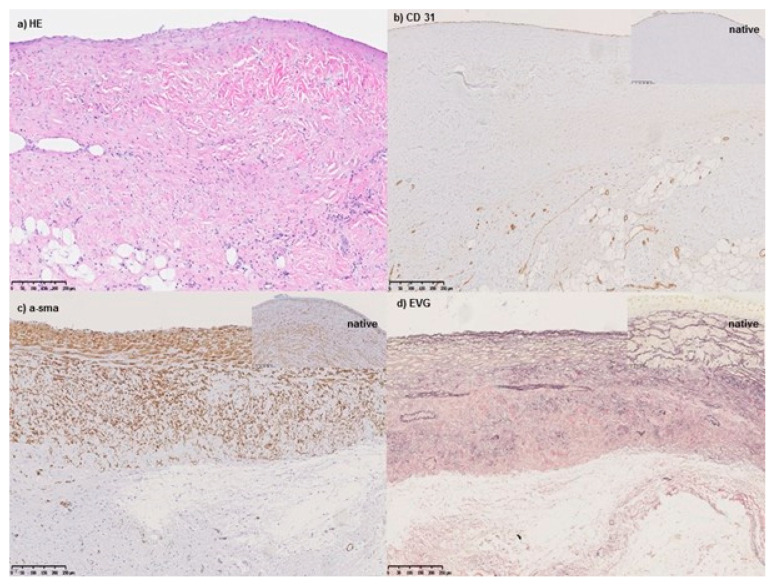
Showing histological results of the conduit of animal #12, which was sacrificed due to infected endocarditis. Routine HE (**a**), immunohistological (**b**,**c**), and special staining (**d**) of the conduit distal to the valve leaflets. The native vessel staining pattern is shown in the insets. (**b**) Demonstrates endothelial cell differentiation at the luminal surface as well as staining of small vessels within the wall. (**c**) Demonstrates smooth muscle differentiation, giving the vessel contractile properties. (**d**) Shows elastic fiber deposition and an ability to passively respond to pressure changes.

**Figure 5:**
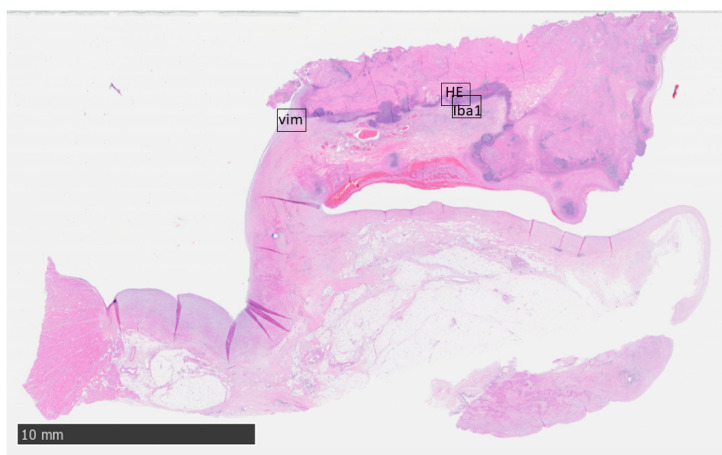
Showing histological overview of leaflets of animal #12, which was terminated due to infected endocarditis. Thickened and distorted valve with suppurative inflammation, bacterial proliferation, and fibrin deposition. Rectangles mark subsequent high-power views (Supplementary Material; Figures S2–S5).

One animal (#18) of the cohort died unexpectedly 23 days after surgery. Histology revealed an aspiration pneumonia as the cause of death. Two animals (#10 and #12) were euthanized before reaching the scheduled study endpoint, 18 and 256 days after surgery, respectively. Both animals presented with intermittent-to-persistent fever and depressed general condition and were treated with antibiotics (enrofloxacin for seven days, and sulfadoxin-trimethoprim and piperacillin for six days) In these animals, infective endocarditis was verified by clinical course, echocardiography, histological, and microbiological examinations. However, the white blood cell count was not significantly elevated. Transesophageal echocardiography showed in both cases severe conduit valve regurgitation and thickening of all leaflets. Histologic evaluation revealed overt infective endocarditis: the conduit leaflets were ulcerated, markedly expanded, and partially effaced by fibrin and exhibited suppurative inflammation with myriad admixed bacterial colonies. Enterobacter cloacae was identified in blood cultures, but contamination at the time of sampling could not be ruled out here. The wall of the conduit was intact and uninvolved and populated predominantly by spindle cells (vimentin-positive), indicating a mesenchymal stromal population. Further stains showed tissue differentiation into multiple components. Strong positive staining for α-sma indicated smooth muscle differentiation; the cellular organization of the vessel wall of the valve conduit did not show the same layering but did show comparable cellular orientation to that found in the native vessel. Elastic fibers were numerous, though finer than in native valve tissue. The conduit exhibited normal endothelialization (as demonstrated by CD31 positive staining), while the outer aspect was merged with pre-existing adipose and connective tissue. Macrophages were present in the vessel wall of the valve conduit, but also found in the native surrounding pulmonary artery, suggesting an inflammatory response due to infective endocarditis rather than to the valve conduit per se. Valved conduit histology stains of animal #12 are depicted in Figure 4. The valve leaflets were thickened and with suppurative inflammation, bacterial proliferation, and fibrin deposition (Figure 5) seen in HE (Supplementary Data; Figure S2) and Iba-1 (Supplementary Data; Figure S3) staining. Vimentin staining (Supplementary Data; Figure S4) showed positive spindle-shaped cells, indicating stromal repopulation and abruptly end top right in the area of fibrin deposition. Further mature bone, mineralized periphery, and central adipose recapitulating the marrow cavity were found (Supplementary Data; Figure S5).

## 4. Discussion

Our study group established a growing animal model to evaluate a TE valved conduit, which is easily self-constructed out of a commercially available de-cellularized porcine small intestinal submucosal ECM patch (CorMatrix^®^) [7]. Some authors tested a similar approach with shorter follow-up until eight months [12,13,14]. Our mid-term results after one year underline the necessity for a much longer follow-up to answer further questions, i.e., at what time point the scaffold vanishes, how long the phase of cellular repopulation lasts, and what are the risk factors leading to valve calcification. For example, we saw mild calcification in only one animal at 9 months (11%; one in nine) while this was present in 50% (four of eight) at 12 months. It would be critical to determine how likely these mild calcifications are to progress to a level impairing valve function. One other interesting point to investigate is the level of agreement, or any discrepancies, between echocardiographic results and histological findings. Our preliminary findings suggest that a 24-month follow-up would be able to shed more light on many of these issues. Of note, our results indicate a mid-term successful functionality of TE valve conduits at one-year follow-up. Our study revealed few limitations, which have also been described by others [15,16,17,18,19,20].

### 4.1. Mortality in a Long-Term Large Animal Model

Overall, we successfully implanted a three-cusp pulmonary valve conduit made entirely from biodegradable material in a chronic growing lamb model with a follow-up period of 12 months. Despite a perioperative mortality rate of 36.8% (7 of 19), the mid-term survival of the remaining animals was 75% (9 of 12). Nevertheless, we experienced one unexpected sudden death due to aspiration pneumonia in a lamb. Two further animals suffered from infective endocarditis despite antibiotic treatment and were euthanized according to study protocol regulations. The early (18 days after surgery) case of infective endocarditis may be due to perioperative bacterial contamination, while the reason for the late (256 days after surgery) case of infective endocarditis remains unclear, as the animal had been out at grass and clinically healthy for over eight months. It is suspected that possible imperfections of surface endothelialization may have increased thrombogenicity of the valves and predisposed to surface colonization of unrelated bacteremia. While native infective endocarditis is not reported very often in sheep [21,22], prosthetic valve infective endocarditis in the sheep model has been noted by other investigators as well [23,24,25,26]. Bernal et al. discussed that complete sterile preparation of the surgical field in sheep is sometimes difficult to assure due to the thick wool. The early onset of infective endocarditis in one animal may result from perioperative bacterial contamination of the valve conduit. However, targeted antibiotic treatment did not resolve endocarditis. It remains open how far the valve conduit itself is sensitive to bacterial adhesion and infection despite the potential of the TE scaffold for remodeling to “normal” valve tissue.

### 4.2. Predisposition or Resistance to Infection

Despite these changes in the valve with respect to risk for infection, histology showed abundant smooth muscle cells with elastic fibers within the valved conduit wall, indicating remodeling with physiologic tissue properties such as vessel valve wall stretching and elastic recoil. The endothelialization included cells of quiescent morphology as a key point for reducing the likelihood of thrombotic events, with vessel wall injury and exposure of underlying stroma cells being one factor of Virchow’s triad for thrombosis. Clinical signs of thromboembolic events were not observed.

### 4.3. Valve Hemodynamics

Our study found the hemodynamic properties of the valve conduit after one year to be excellent in respect to stenotic valve changes, while some authors reported gradual functional deterioration of valve conduit in mid-term follow-up, when used as pulmonary valve reconstruction [15] or a failure to remodel in a structured and anatomical fashion in an arterial environment [16]. Our results showed normal hemodynamic function of the implanted valve conduit determined by repeated echocardiographic Doppler flow profiles over one year. Valve calcification developed over time in 5 of 12 animals (41.6%). Even when calcification occurred early (in one animal starting at 3 months after surgery), we found only a low impact on the hemodynamic function of the implanted valved conduit. Other mechanisms for valve regurgitation including valve leaflet prolapse may coexist and may be the cause of functional deterioration. Therefore, further follow-up of functional properties of the valve conduit up to at least a two-year post-surgery timepoint is required.

### 4.4. Conduit Growth

The increase in body weight of our animals overtook the increase in conduit valve annulus diameter. Accordingly, Miller et al. reported a slower increase in diameter of a pulmonary valve replacement conduit compared to the native tissue in a porcine model with a concomitant increase in the peak systolic gradient at six months [14]. In our study, we did not observe an increase in blood flow velocity across the conduit. During the twelve-month follow-up, no animal showed clinical, echocardiographic, or laboratory signs of a small for size conduit mismatch. In fact, we did not observe a peak systolic gradient above 31 mmHg, which was measured in an animal with moderate valve regurgitation and, therefore, may have been slightly overestimated. Lambs are considered fully grown after six months. In the absence of structural deterioration, continuing growth of the valved conduit may even result in a reduction in the valve pressure gradient during the following twelve months of the study.

### 4.5. Hemolysis

Hemolysis is well known as a complication of prosthetic heart valves and is usually associated with structural deterioration. LDH values increased after surgery compared to pre-operative values but remained only slightly elevated throughout the follow-up period compared to healthy published cohorts [10,11]. In addition, hemoglobin values were stable over time, and no animal suffered clinically from hemolytic anemia.

By using a chronic growing lamb model, we aimed to detect early occurrence of structural changes and calcification of the valve conduit. In contrast to others [14,16], after 12 months, we frequently found calcification (detected by echo, CT, and/or histology), but it was rather mild with a low impact on valve conduit hemodynamic performance. Here, long-term data are needed.

## Figures and Tables

**Table 1:** Demographics, perioperative data, and cause of death of seven animals who did not survive the perioperative phase. ID: Identification number, Kg: kilograms, mm: millimeters, POD: post-operative day, OR: operation, and CPB: cardiopulmonary bypass.

Animal ID	Weight Pre-Operative [kg]	Conduit Diameter [mm]	Death Intraoperatively	Death Post-Operatively [POD]	OR Time [min]	CPB Time [min]	Cause of Death
#7	29	20	No	1	405	270	Low cardiac output
#8	27	18	Yes	-	240	54	Diffuse hemorrhage
#9	24	18	No	1	195	74	Acute hemorrhage from femoral artery (puncture site of arterial line) in the stable
#11	35	20	No	0	163	63	Low cardiac output
#13	32	18	No	1	185	52	Pre-operatively reduced LV function, difficult weaning from CPB, use of inotropes, died in heart failure
#19	30	18	No	1	175	45	Pericardial tamponade with immediate re-operation; death secondary to anemia and hypoproteinemia with no possibility of transfusion
#21	28	18	Yes	-	205	117	Weaning failure from CPB
Median (range)	29 (24–35)	18 (18–20)	-	1 (0–1)	195 (163–405)	63 (45–270)	-

**Table 2:** Demographics, perioperative data, and valve conduit calcification of 12 animals who survived the perioperative phase. ID: Identification number, Kg: kilograms, mm: millimeters, OR: operation, CPB: cardiopulmonary bypass, HCT: hematocrit, FU: follow-up, n.a.: not applicable, and POD: post-operative day. * Calcifications were verified by echocardiography.

Animal ID	Weight Pre-Operative [kg]	Conduit Implantation Diameter [mm]	Outcome	OR Time [min]	CPB Time [min]	HCT before CPB [%]	HCT after CPB [%]	Weight at Last FU [kg]	Valve Conduit Calcification * (at Last FU)
#5	33	20	Ongoing	150	70	22	20	55	Mild (12 months)
#6	30	20	Ongoing	162	71	20	18	38	None (12 months)
#10	27	20	Died before endpoint	160	56	24	21	n.a.	None (POD 18)
#12	35	20	Died before endpoint	183	52	22	21	59	None (POD 256)
#14	33	18	Sacrificed per protocol at 12 months	165	44	25	21	48	None (12 months)
#15	36	16	Ongoing	185	61	21	18	53	Mild (12 months)
#16	34	18	Sacrificed per protocol at 9 months	113	40	21	20	48	Mild (9 months)
#18	36	22	Died before endpoint	192	80	23	10	n.a.	None (POD 23)
#17	38	18	Ongoing	104	37	24	21	48	None (12 months)
#20	30	18	Ongoing	151	40	20	19	60	None (12 months)
#22	28	18	Ongoing	125	34	20	15	53	Mild (12 months)
#23	29	18	Ongoing	110	31	16	15	66	Mild (12 months)
Median (range)	33 (27–38)	18 (16–22)	-	155.5 (104–192)	48 (31–80)	21.5 (16–25)	19.5 (10–21)	53 (38–66)	

**Table 3:** Mean gradient across the pulmonary valve for those animals that survived the first month. Valve regurgitation is labeled grade 0 for absent, grade 1 for trivial, grade 2 for mild, grade 3 for moderate, and grade 4 for severe. POD: post-operative day. * Calcifications were verified by echocardiography.

Animal ID		1 Month Post-op	3 Months Post-op	6 Months Post-op	9 Months Post-op	12 Months Post-op	Valve Conduit Calcification * (at Last FU)	Outcome
#5	Mean gradient/regurgitation	3/0	3/0	3/0	2/0	2/1	Mild (12 months)	Ongoing
#6	Mean gradient/regurgitation	10/0	4/0	4/0	3/0	3/1	None (12 months)	Ongoing
#12	Mean gradient/regurgitation	2/1	4/1	6/0			None (POD 256)	Unexpected death
#14	Mean gradient/regurgitation	4/3	6/0	3/2	3/4	¾	None (12 months)	Sacrificed per protocol at 12 months
#15	Mean gradient/regurgitation	4/1	9/0	2/0	2/0	3/0	Mild (12 months)	Ongoing
#16	Mean gradient/regurgitation	2/0	14/1	5/0	15/3		Mild (9 months)	Sacrificed per protocol at 9 months
#17	Mean gradient/regurgitation	3/0	5/0	2/1	2/1	2/0	None (12 months)	Ongoing
#20	Mean gradient/regurgitation	1/1	3/1	3/1	1/1	12/1	None (12 months)	Ongoing
#22	Mean gradient/regurgitation	12/1	4/2	11/1	10/1	12/2	Mild (12 months)	Ongoing
#23	Mean gradient/regurgitation	6/0	3/0	3/0	7/1	5/1	Mild (12 months)	Ongoing

**Table 4:** Course of laboratory values over time. POD: Post-operative day, LDH: lactate dehydrogenase, HB: hemoglobin, and WBC: white blood cell count. Only animals were included that survived first 30 post-operative days.

		1 Month after Surgery	3 Months after Surgery	6 Months after Surgery	9 Months after Surgery	12 Months after Surgery
LDH, U/L	Median (range)	1152 (882–1397)	1151 (1079–1329)	1182 (1013–1448)	1248 (967–1369)	1121 (951–1514)
HB, g/L	Median (range)	110 (88–115)	114 (107–133)	111 (89–134)	114 (103–125)	108 (93–113)
WBC, 10^9^/L	Median (range)	7.6 (8.82–9.95)	7 (6.8–8.71)	5.7 (5.22–7.6)	6,1 (5.6–8.84)	6.7 (6.5–9.45)

## Data Availability

As this is an ongoing study, data is not further data is not public available at the moment.

## Supplementary Materials

The following supporting information can be downloaded at: https://www.mdpi.com/article/10.3390/jcdd11060179/s1, Figure S1. Self-constructed valved conduit out of a commercially available de-cellularized porcine small intestinal submucosal extracellular matrix biologic scaffold. Figure S2. High power view of rectangle marked area of Figure 5 in HE staining. It shows central basophilic stippling represents a bacterial colony, deeply basophilic clumped material beneath represents degenerate neutrophils whilst the eosinophilic acellular fibrillar material is fibrin. Figure S3. High power view of rectangle marked area of Figure 5 in Iba1 staining. It shows extensive infiltration, abruptly negative in the area of fibrin deposition top left. Figure S4. High power view of rectangle marked area of Figure 5 in Vimentin staining. It shows positive spindle shaped cells indicate stromal repopulation, abrupt end top right in area of fibrin deposition. Figure S5. Same conduit as shown in Figure 5, representing a sheep died due to endocarditis. HE staining shows the wall of the conduit with mature bone; mineralised periphery and central adipose recapitulating the marrow cavity.
