# Supplementary material for: One-Year Outcome of an Ongoing Pre-Clinical Growing Animal Model for a Tissue-Engineered Valved Pulmonary Conduit
Source: J Cardiovasc Dev Dis. 2024 Jun 12;11(6):179. doi: 10.3390/jcdd11060179 (PMC11204005; doi:10.3390/jcdd11060179)
Supplement: Supplementary file 1 [file jcdd-11-00179-s001.zip › jcdd-2858136-supplementary.pdf]

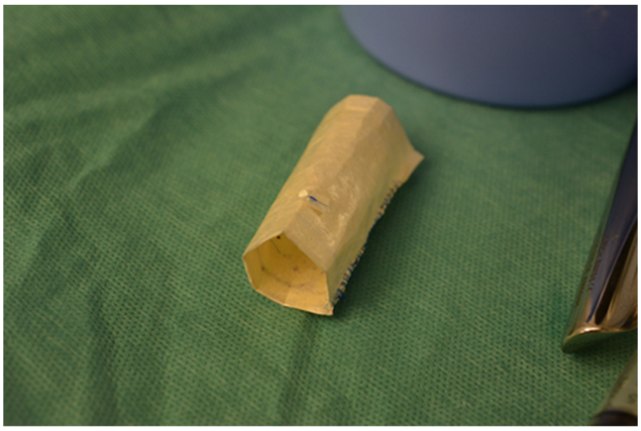

Figure S1. Self-constructed valved conduit out of a commercially available de-cellularized porcine small intestinal submucosal extracellular matrix biologic scaffold.

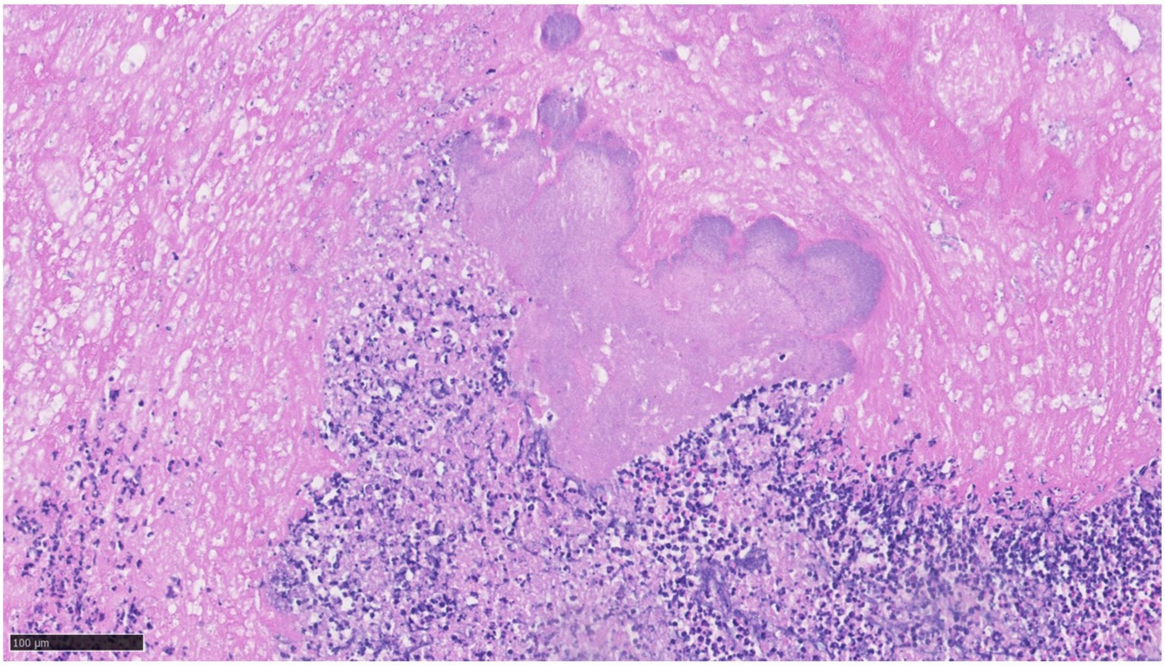

Figure S2. High power view of rectangle marked area of Figure 5 in HE staining. It shows central basophilic stippling represents a bacterial colony, deeply basophilic clumped material beneath represents degenerate neutrophils whilst the eosinophilic acellular fibrillar material is fibrin.

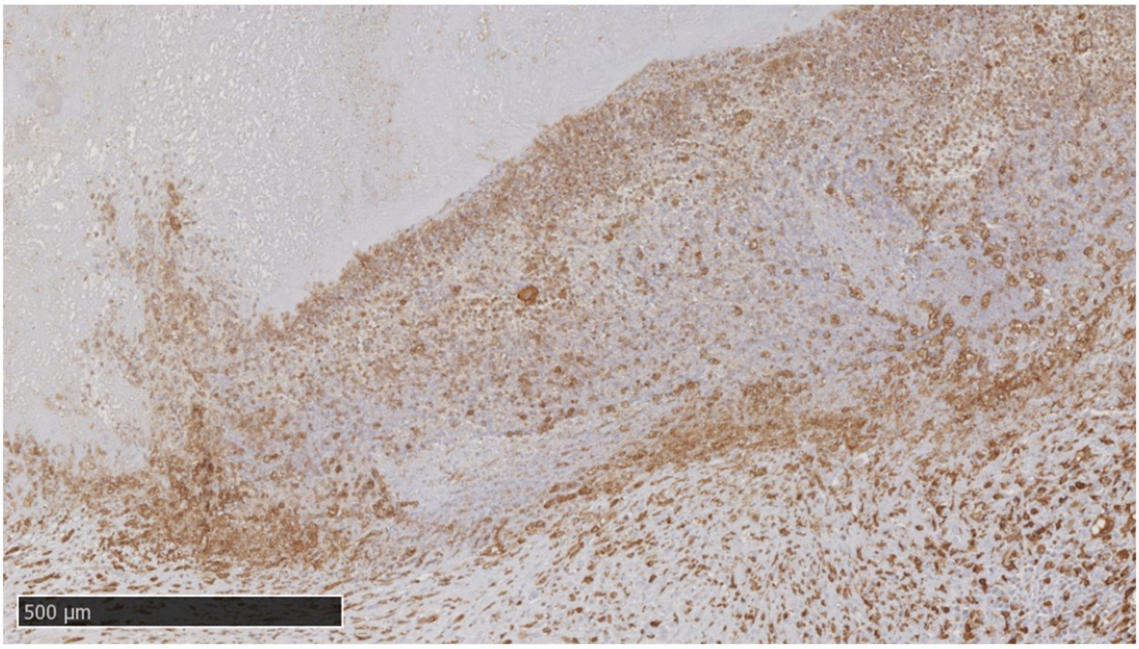

Figure S3. High power view of rectangle marked area of Figure 5 in Iba1 staining. It shows extensive infiltration, abruptly negative in the area of fibrin deposition top left.

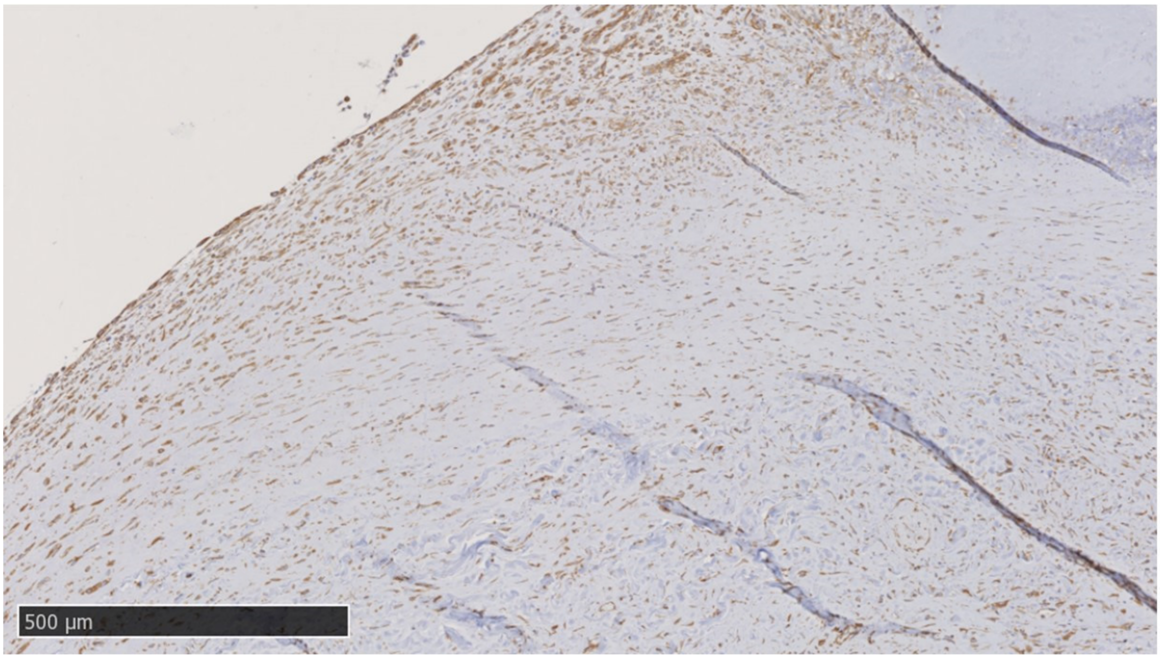

Figure S4. High power view of rectangle marked area of Figure 5 in Vimentin staining. It shows positive spindle shaped cells indicate stromal repopulation, abrupt end top right in area of fibrin deposition.

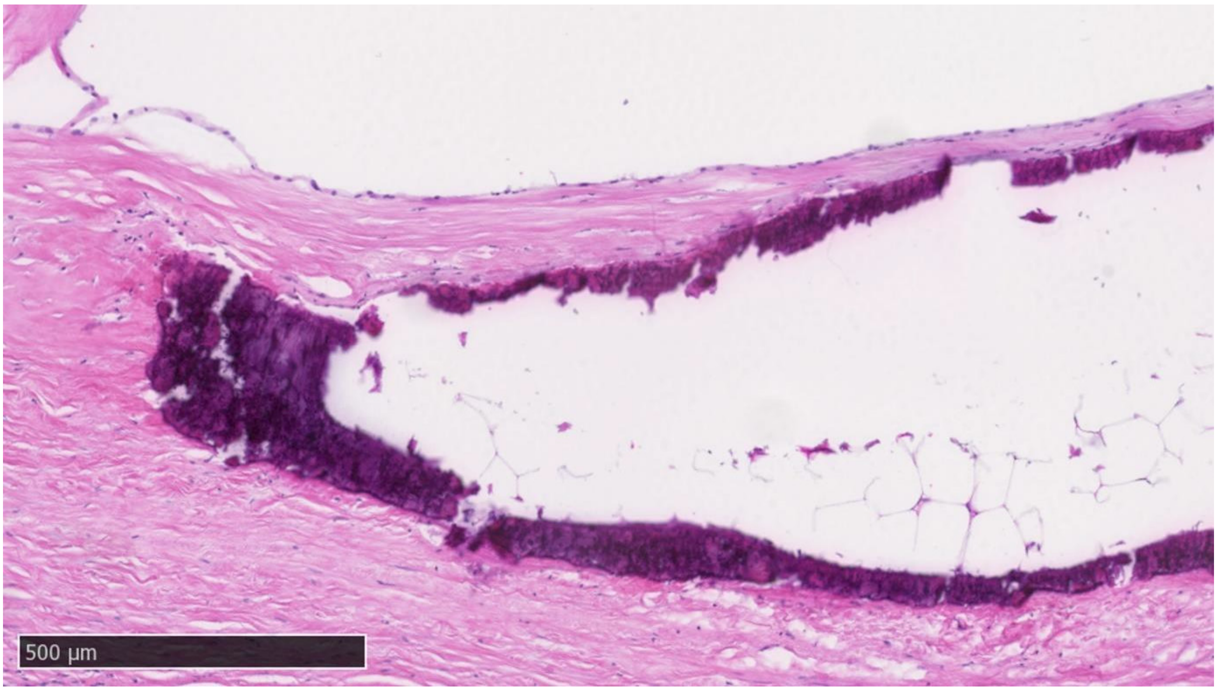

Figure S5. Same conduit as shown in Figure 5, representing a sheep died due to endocarditis. HE staining shows the wall of the conduit with mature bone; mineralised periphery and central adipose recapitulating the marrow cavity.
